# Immunoassay-based quantification of full-length peptidylglycine alpha-amidating monooxygenase in human plasma

**DOI:** 10.1038/s41598-023-37976-3

**Published:** 2023-07-04

**Authors:** Yulia Ilina, Paul Kaufmann, Olle Melander, Michaela Press, Katrin Thuene, Andreas Bergmann

**Affiliations:** 1 PAM Theragnostics GmbH, Neuendorfstr. 15A, 16761 Hennigsdorf, Germany; 2 Department of Clinical Sciences Malmö, Lund University, 205 02 Malmö, Sweden; 3 Department of Emergency and Internal Medicine, Skåne University Hospital, Malmö, Sweden

**Keywords:** Diagnostic markers, Predictive markers

## Abstract

A one-step sandwich chemiluminescence immunometric assay (LIA) was developed for the quantification of bifunctional peptidylglycine-α-amidating monooxygenase (PAM) in human plasma (PAM-LIA). PAM is responsible for the activation of more than half of known peptide hormones through C-terminal α-amidation. The assay employed antibodies targeting specific catalytic PAM-subunits, peptidylglycine alpha-hydroxylating monooxygenase (PHM) and peptidyl-alpha-hydroxyglycine alpha-amidating lyase (PAL), to ensure detection of full-length PAM. The PAM-LIA assay was calibrated with a human recombinant PAM enzyme and achieved a detection limit of 189 pg/mL and a quantification limit of 250 pg/mL. The assay demonstrated good inter-assay (6.7%) and intra-assay (2.2%) variabilities. It exhibited linearity when accessed by gradual dilution or random mixing of plasma samples. The accuracy of the PAM-LIA was determined to be 94.7% through spiking recovery experiments, and the signal recovery after substance interference was 94–96%. The analyte showed 96% stability after six freeze–thaw cycles. The assay showed strong correlation with matched EDTA and serum samples, as well as matched EDTA and Li-Heparin samples. Additionally, a high correlation was observed between α-amidating activity and PAM-LIA. Finally, the PAM-LIA assay was successfully applied to a sub-cohort of a Swedish population-based study, comprising 4850 individuals, confirming its suitability for routine high throughput screening.

## Introduction

**Figure 1:**
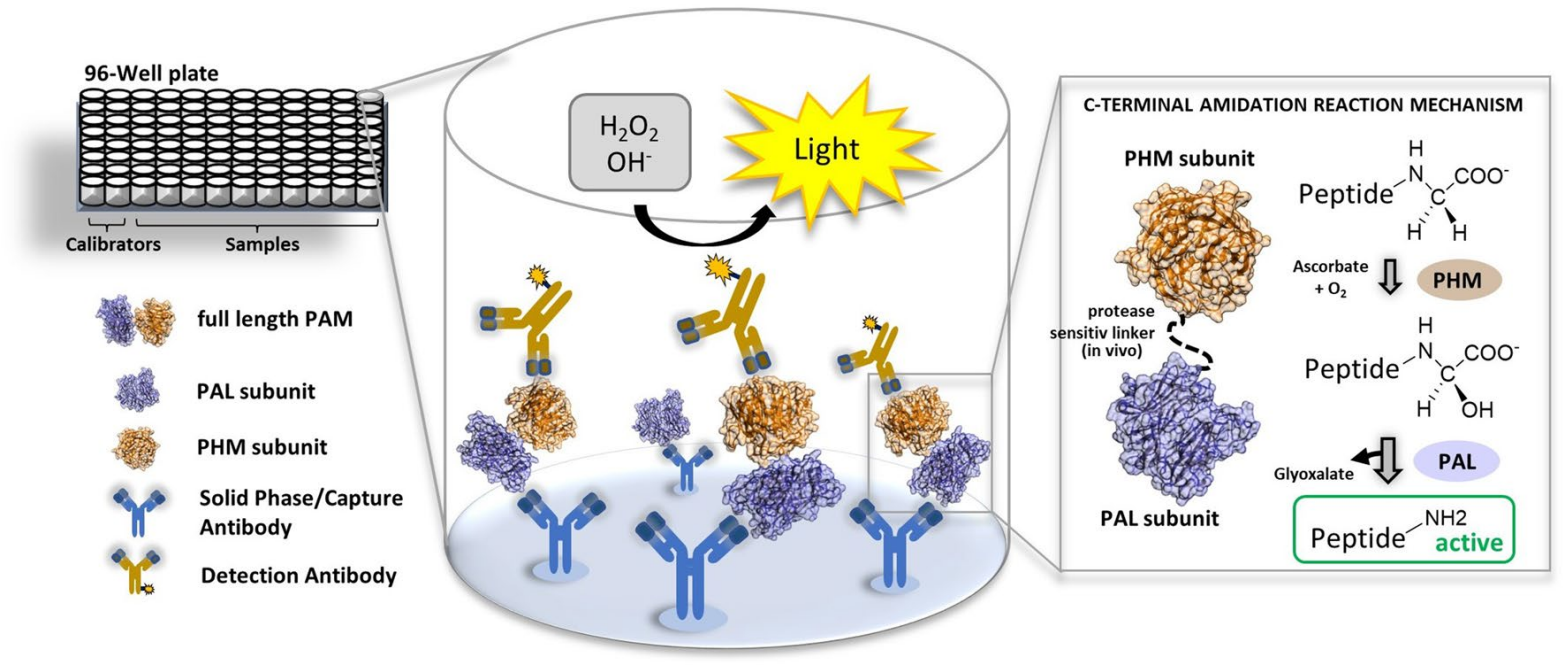
Schematic representation of PAM-LIA assay. Detection of full-length PAM is achieved in one-step protocol by capturing the PHM-subunit and detecting the PAL subunit, depicted as surfaces in navy blue and orange, respectively. The simplified reaction mechanism of C-terminal α-amidation is shown in the right inset.

One of the major groups of intercellular agents for cell-to-cell communications are peptides, acting both as paracrine and endocrine signaling agents and playing a role in a vast range of physiological and pathophysiological processes. All peptide hormones are synthesized as large protein precursors and require well-orchestrated series of proteolytic processing and post-translational modifications in order to gain their full biologic activity. One such modification is C-terminal α-amidation, a reaction essential for half of the known peptide hormones and it is catalyzed by a bifunctional enzyme peptidylglycine alpha amidating monooxygenase (PAM)^1,2^. PAM is a monopeptide, comprising two functional subunits with distinct catalytic activities, a peptidylglycine alpha-hydroxylating monooxygenase (PHM) and a peptidyl-alpha-hydroxyglycine alpha-amidating lyase (PAL) (Fig. 1, inset right)^3,4^. Both catalytic domains work sequentially in order to convert a peptide intermediate into its active alpha-amidated form. In the first step, C-terminal glycine of the peptide hormone precursor is α-hydroxylated by PHM upon binding to its ascorbate reduced copper active site^5,6^. In the next step, zinc-dependent PAL subunit cleaves the glyoxylate from the peptidyl-α-hydroxyglycine previously generated by PHM^7^, which leads to a formation of the c-terminal α-amine group and full activation of the peptide hormone (Fig. 1).

Encoded by a single copy gene with more than 160 kb of DNA comprising 25 exons, human PAM exists in at least six different isoforms, including membrane bound and soluble variants, as well as single monofunctional PHM and PAL enzymes^8^. Along with alternative splicing, the latter can also be attributed to a tissue-specific endoproteolytic cleavage of protease sensitive linker between two catalytic domains. Initially isolated from the bovine neurointermediate pituitary lobe^9,10^, the majority of the PAM activity was associated with separate PHM and PAL catalytic domains. In contrast, in bovine atrium and rat medullary thyroid carcinoma purified PAM was present as an intact bifunctional enzyme of a 113-kDa and 75-kDa, respectively^11–13^.

PAM is indispensable for life and is the only known enzyme to catalyze the C-terminal α-amidation^4^. It was demonstrated, that PAM double knock-out in mice and fruits flies is lethal in the first week of gestation or early larval stage, respectively, leading to a complete depletion of amidated peptide hormones^14,15^. Furthermore, the Drosophila nemy mutants, which encode defective redox enzymes (cytochrome B561 homologue), a required cofactor for PHM activity, showed decreased levels of amidated peptides and learning and memory deficits^16^. PAM is expressed in most mammalian cell types with the highest PAM activity in pituitary and hypothalamus^2,17^. In human, the modulation in PAM activity has long been linked to the presence or potential development of the diverse human pathologies^4,18^. The increased α-amidating activity was found in several tumors, including the medullary thyroid carcinoma, neuroendocrine and pancreatic endocrine tumors, and also was associated with multiple sclerosis, post-polio syndrome and many others^19–22^. Multiple PAM mutations resulting in reduced activity were shown to be associated with an increased risk of type 2 diabetes, possibly by affecting insulin granule packaging and release from β-cells^23–25^.When measured in CSF of the Alzheimer’s patients, the PAM activity was significantly reduced in comparison to the healthy individuals^26^. According to the recent findings, also in plasma the reduced PAM activity was associated with higher risk of AD development 7 years prior the diagnosis^27^. The elevated α-amidating activity was correlated with higher incidence of heart failure and heart fibrillation, as well as untreated systolic and diastolic hypertension^28^. In addition to its primary function of catalyzing the C-terminal amidation of numerous peptide hormones, Bäck et al. has demonstrated that PAM is also necessary for the formation of atrial secretory granules^29^. Studies using knockout mice that lack cardiomyocyte PAM have shown a decrease in the content of atrial natriuretic peptide (ANP) and brain natriuretic peptide (BNP) despite the fact that these peptides do not require α-amidation. Thus, due to its essential role in regulation of versatile physiological and pathophysiological functions, PAM was suggested to be significant therapeutic target and biomarker for various human clinical conditions^4^.

To this day the most common way to quantify PAM enzyme is by measuring its activity. Most of these methods are unapplicable for the routine large-scale applications, since the quantification of the α-amidated products require time-consuming analytical chromatography or other substrate/product separation methods (e.g. with ethyl-acetate) for each sample^20,30–34^. One other drawback is the utilization of the synthetic radiolabeled tri- or quadropeptides as a substrate^20,30–34^. To our knowledge, there are only few activity assays, which utilize the unmodified naturally occurring full-length peptide hormone precursor as a substrate^28,35^. Finally, the quantification of PAM activity is usually accessed under optimal co-factor concentration, such as copper and ascorbate, which may differ from their actual concentrations in vivo and possibly artificially elevate the determined PAM activity. While the activity-based quantification of PAM has been explored, the information regarding the direct measurement of PAM enzyme concentration is scarce in the existing literature^36^. Commercially available ELISA assays for PAM quantification lack information regarding the specificity of the capturing and detecting antibodies. This limitation becomes particularly disadvantageous when attempting to distinguish between the full-length enzyme and single subunits. Another drawback is the lack of information addressing the correlation between PAM enzyme activity and its concentration, which might hinder the transferability of findings gained from activity-based measurements into quantitative estimations of enzyme load.

Here we present the one-step chemiluminescence immunometric assay, which allows to determine the concentration of bifunctional PAM enzyme in human plasma (PAM-LIA). Conformational capture and developing antibodies were directed against PHM and PAL subunit, respectively, enabling the detection of the full-length enzyme variant. The PAM-LIA assay is designed in 96-well microtiter plate-based format, requires a small sample volume of 20 µL for single determination, short incubation time and operates within a wide calibration range of 0.25–723 ng/mL. Our immunoassay enables high-throughput screening of a large number of samples within a short timeframe. To demonstrate the efficiency of PAM-LIA assay as well as possible application of PAM as a predictive biomarker for several cardiovascular events, we measured PAM concentration in a sub-cohort of 4850 study subjects from a Swedish population-based study, the Malmo Preventive Project.

## Results

PAM-LIA is a one-step high throughput microtiter plate-based chemiluminescence immunometric assay for the quantitative detection of full-length PAM in human with the capture and detection antibodies being specifically directed against PAL and PHM subunits, respectively (Fig. 1). No cross-reactivity was measured when a single recombinant PAL or PHM were used as the analytes. Synthetic bifunctional PAM lacking the protease sensitive linker between PAL and PHM subunits was used as the calibrator, covering a linear concentration range up to 723.4 ng/mL. The PAM-LIA reached its saturation plateau above 1000 ng/mL, and a high-dose hook effect started at concentrations above 6 μg/mL.

**Figure 2:**
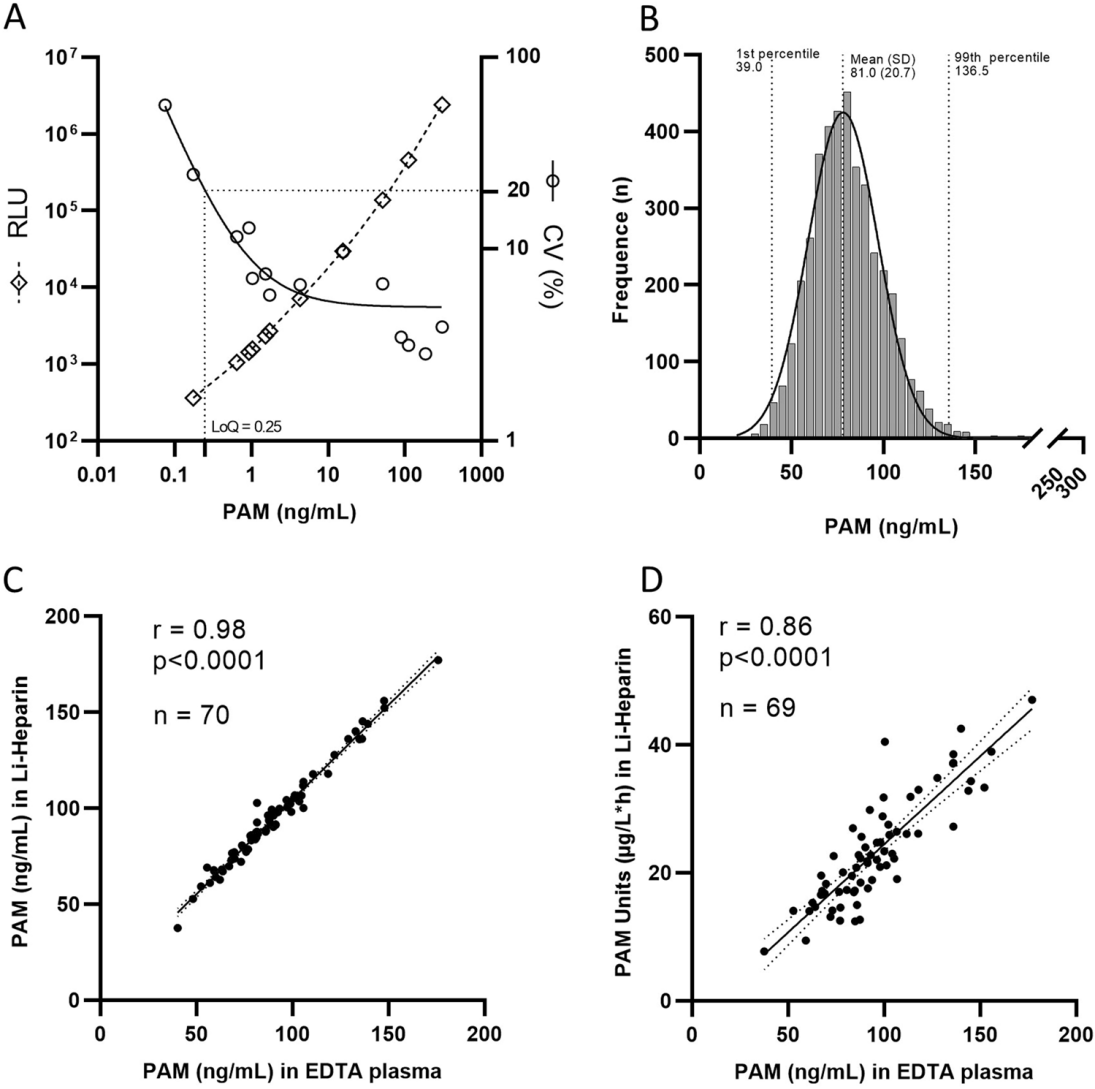
(**A**) The dose–response curve of the calibrator (squares) and interassay precision profile (circles). LoQ was calculated from the precision profile function (solid line) at a CV value equal to 20%. Each point represents a mean of 16 measurements; (**B**) Frequency distribution of PAM (ng/mL), measured in serum in 4850 individuals (sub-cohort of the Malmö Preventive Project). The median PAM concentration and the 95% CI (dotted line) were calculated from the fitted normal distribution curve (solid line). The data failed the D’Angostino and Pearson normality test. Non-parametric Spearman correlation of PAM concentration, determined in the PAM-LIA assay, measured between matched EDTA plasma and Li-Heparin (**C**). The correlation between PAM concentration (in ng/mL), determined in the PAM-LIA assay, and PAM amidating activity (in µg/L*h) (**D**), measured as described in Kaufmann et al. 2021. n being the number of matched pairs, r representing the correlation coefficient and *p*-value for statistical significance. The 95% confidence interval is shown as dotted lines.

Technical performance of the PAM-LIA was summarized in Table 1. The average intra-assay CV was 2.2% [1.3–3.8%] and the average inter-assay CV was 6.7% [2.8–12.9%]. The LOD and LOQ were 189 pg/mL and 250 pg/mL, respectively, as interpolated from a dose–response curve (Fig. 2A). The assay linearity was access by dilution and by mixing. In the first case, the average deviation between the measured and targeted concentrations for the sample with starting PAM concentration of 91.2 ng/mL, 323.5 ng/mL and 684.7 ng/mL were 13.2% [8.9–7.9%], 1.2% [3.7–8.2%] and 5.2% [0.4–8.8%], respectively (Fig. S1A–C). In the second case, the determined concentration of PAM deviated from the expected concentration on average by 4.9% [0.7–10.2%], calculated from n = 19 pool samples (Fig. S1D). The accuracy of the PAM-LIA assay was determined by spiking of analyte-depleted EDTA plasma with a known concentration of recombinant PAM and was in the range of 90.3–99.2% (Fig. S1E). All assay characteristics fulfilled the acceptance criteria of ± 20% CV.

**Table 1:** Analytical performance characteristics of PAM-LIA assay.

Calibrator range	0–723.4 ng/mL
High-dose hook	6000 ng/mL
Temperature operational range	18–32 °C
Limit of detection	189 pg/mL
Limit of quantification	250 pg/mL
Recovery (MW) from expected (%) ± recovery range (%)	
Inter-assay CV	6.7% [2.8–12.3%]
Intra-assay CV	2.2% [1.3–3.8%]
Linearity (dilutional recovery)	2.0–91.2 ng/mL
	86.8% [82.1–91.1%]
	1.2–323.5 ng/mL
	98.8% [91.8–103.7%]
	1.2–694.7 ng/mL
	94.8% [91.2–100.4%]
Linearity (mixing recovery)	96.5% [86.6–109.5%]
Accuracy (spiking recovery)	94.7% [90.3–99.2%]
Analytical specificity (substance interference study)	Low pool samples: 94% [88–103%]
	High pool samples: 96% [92–101%]
Ex vivo analyte stability (freeze–thaw analysis)	Average (± SD) % recovery after up to 6 freeze–thaw cycles (averaged from 6 samples): 96% ± 1%
Correlation studies	matched EDTA/Li-Heparin: r = 0.98 *p* < 0.0001
	matched EDTA/serum: r = 0.84 *p* < 0.0001
	PAM amidating activity/PAM concentration: r = 0.86 *p* < 0.0001

Confounding factors used for the substance interference studies are listed in the Table S1. These included typical plasma components, analgesics, anticoagulants, antibiotics, antibacterial and anti-inflammatory agents, antioxidants and others. The average signal recovery in two EDTA plasma pools after supplementation with confounding factors was 94% [88–103%] and 96% [92–101%] for low (contained 54.7 ng/mL endogenous PAM) and high (spiked with 364.6 ng/mL recombinant PAM) EDTA plasma pools, respectively.

The ex vivo analyte stability in six native EDTA plasma samples, collected from healthy reported individuals, was determined (Fig. S2A). The average concentration of full-length PAM was 88.2 ng/mL [74.7–102.5 ng/mL]. After the sixth freeze–thaw cycle the concentration of PAM reduced on average by 4.3% [2.2–7.1%], when compared to the PAM concentration measured in the non-frozen samples.

The accepted operational temperature range of PAM-LIA assay was determined between 18 °C and 32 °C (Fig. S2B). The PAM concentrations, determined at 18 °C, 20 °C, 25 °C, 28 °C or 32 °C, yielded 96.2% [92.3–100.6%] of the PAM value determined at 22 °C, which was the operational temperature of the standard assay protocol.

Since assay parameters such as precision, linearity and analyte stability were determined in EDTA plasma, it is a preferred matrix for the PAM-LIA assay. However, a strong correlation of 0.98 (*p* < 0.0001) and 0.84 (*p* < 0.0001) between matched Li-Heparin and EDTA plasma (Fig. 2C) and matched serum and EDTA plasma (Fig. S3A), respectively, was observed. Additionally, a significant correlation between the plasmatic amidating activity in Li-Heparin and the PAM concentration measured in matched EDTA plasma was shown. In a small cohort of n = 69, the correlation coefficient was r = 0.86 (*p* < 0.0001) (Fig. 2D). This correlation was further confirmed in a larger cohort of n = 4850 with r = 0.71 (*p* < 0.0001) (Fig. S3B).

In addition to measuring PAM concentrations in human plasma and serum, the assay was found to be suitable for use with rat, porcine and ape plasma. The average PAM concentrations in these samples were determined to be 7.1 ng/mL (measured in a plasma pool of n = 3 animals), 8.42 ng/mL (n = 3) and 143.0 ng/mL (n = 11), respectively.

The distribution of PAM concentration in serum samples from a sub MPP cohort of n = 4850 individuals is presented in Fig. 2B. The mean PAM-LIA value was 81.0 ng/mL [SD = 20.7]. The median PAM concentration was 79.5 ng/mL, with an interquartile range (IQR) of 67.1–93.1 ng/mL. The 10th and 90th percentiles were 56.3 and 107.2 ng/mL, respectively. The 2.5th, 97.5th, and 99th percentiles were 44.7 ng/mL, 124.8 ng/mL, and 136.5 ng/mL, respectively. The normal distribution of PAM concentration failed the test for normality (D’Agostino Pearson omnibus test).

Furthermore, PAM concentration was divided into quartiles and correlated with clinical baseline parameters such as age, BMI, cholesterol, glucose levels, etc. (Table 2). The results of this analysis indicate that there is a significant correlation between increasing levels of PAM and age (*p* < 0.0001), BMI (*p* < 0.0001), systolic (*p* = 0.0002) and diastolic blood pressure (*p* = 0.0019), and triglyceride levels (*p* = 0.0008). However, there were no significant correlations found between gender, glucose, LDL, HDL, and total cholesterol levels and PAM protein.

**Table 2:** Baseline clinical characteristics according to quartiles (Q).

	Q1	Q2	Q3	Q4	*p* value
	n = 1218	n = 1208	n = 1207	n = 1217	
Range (ng/mL)	9.5–67.1	67.2–79.5	79.6–93.0	93.1–242.4	n.d
mean PAM in ng/mL (SD)	56.7 (8.3)	73.6 (3.6)	85.8 (4.0)	108.1 (14.2)	
Age in years (SD)	68.8 (6.3)	69.3 (6.3)	69.6 (6.0)	70.2 (6.4)	< 0.0001
Gender, n male (%)	858 (69.4)	854 (69.0)	845 (68.3)	837 (73.7)	n.d
BMI in kg/m^2^ (SD)	26.7 (3.8)	26.9 (4.0)	27.1 (4.5)	27.5 (4.5)	< 0.0001
Systolic blood pressure in mmHg (SD)	144.0 (20.1)	144.9 (20.0)	145.1 (20.0)	147.5 (21.6)	0.0001
Diastolic blood pressure in mmHg (SD)	83.0 (10.4)	83.3 (10.6)	83.7 (10.6)	84.6 (11.6)	0.0039
Glucose in mmol/L (SD)	5.89 (1.8)	5.80 (1.5)	5.84 (1.3)	5.83 (1.3)	0.0163
Diabetes mellitus, n (%)	203 (16.7)	162 (13.4)	192 (15.9)	207 (17.0)	n.s
LDL in mmol/L (SD)	3.60 (1.0)	3.59 (1.0)	3.63 (1.0)	3.64 (1.0)	n.s
HDL in mmol/L (SD)	1.38 (0.4)	1.40 (0.4)	1.36 (0.4)	1.38 (0.4)	n.s
Cholesterol in mmol/L (SD)	5.53 (1.1)	5.53 (1.1)	5.57 (1.1)	5.60 (1.1)	n.s
Triglyceride in mmol/L (SD)	1.20 (0.6)	1.20 (0.6)	1.26 (0.7)	1.28 (0.6)	< 0.0001

n.a. not applicable, n.s. not significant.

**Figure 3:**
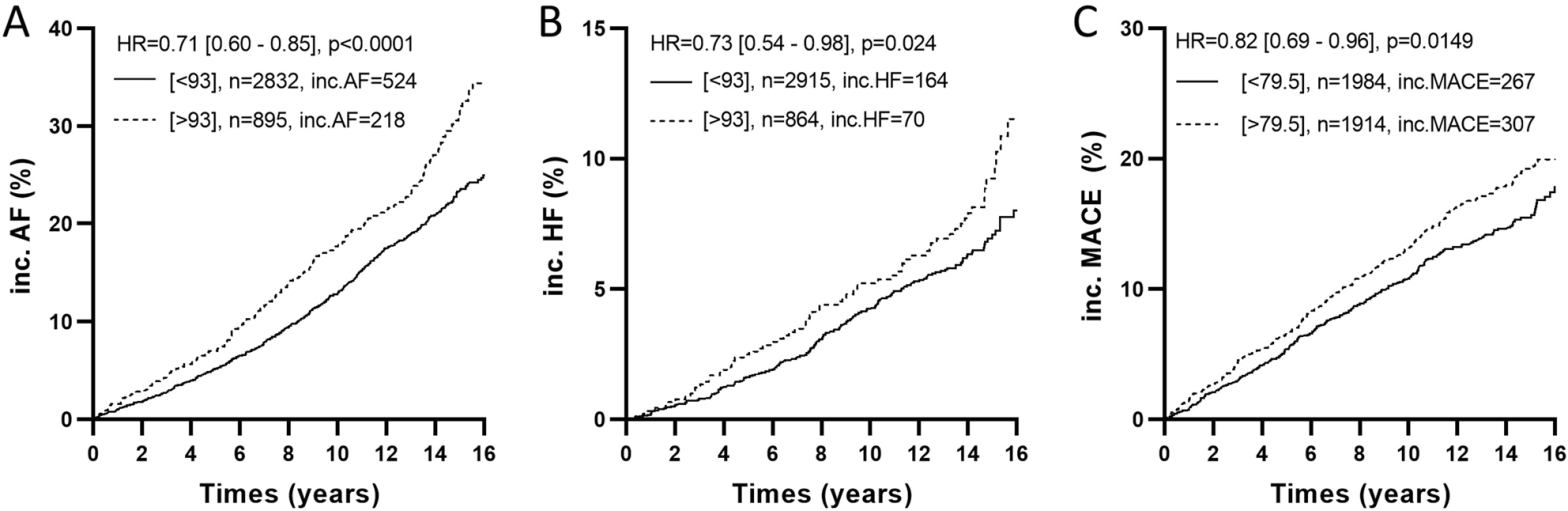
The 16-year follow-up Kaplan–Meier survival curves (Cox Proportional Hazards Model) for low versus high PAM concentration, using cutoffs of 93 ng/mL for incident atrial fibrillation (AF), (**A**) and incident heart failure (HF), (**B**) and 79.5 ng/mL for incident major adverse cardiovascular events (MACE), (**C**). n is the number of subjects (MPP sub-cohort with no history of cardiovascular disease), HR* for hazard ratio (fully adjusted Cox regression with respect to age, gender, BMI, systolic blood pressure, diastolic blood pressure, glucose, diabetes, LDL, HDL, total cholesterol, and triglycerides) and p-values for statistical significance.

Finally, we evaluated the predictive value of PAM-LIA in relation to the incidence of heart failure (HF), atrial fibrillation (AF), and major adverse cardiovascular events (MACE) in a sub cohort of MPP, excluding individuals with pre-existing coronary event and stroke. The results indicated that a PAM concentration above the median value was associated with a higher incidence for HF, AF and MACE. Specifically, individuals with PAM concentrations below 93 ng/mL had a lower incidence of HF (HR = 0.73, *p* = 0.024) and AF (HR = 0.71, *p* < 0.0001), while those with PAM concentrations below 79.5 ng/mL had a lower incidence of MACE (HR = 0.82, *p* = 0.0149) (Fig. 3).

## Discussion

The described one-step sandwich chemiluminescence immunometric assay (PAM-LIA) offers significant advantages over currently applied methods for measuring the level of PAM in bodily fluids. Firstly, most validated methods for measuring PAM levels involve time-consuming and non-scalable methods, which employ the quantification of product formation. Secondly, measuring PAM activity often requires optimal substrate conditions, which may not accurately reflect in vivo conditions. In contrast, the PAM-LIA assay directly measures the protein concentration, which may offer more accurate insights into the in vivo status of the enzyme.

The current assay set-up enables the measurement of the full-length PAM by targeting the PAL subunit through capturing antibodies and detecting the PHM subunit. PAM protein is expressed in various isoforms, including different variants of the full-length protein as well as single subunits, depending on the tissue. To obtain a more comprehensive view of PAM’s expression profile, different permutation of capture and tracer antibodies could be used to target either PHM or PAL alone, either as a single subunit or as part of the full-length enzyme. By combining different assay setups, important insights into the total PAM load in different tissue extracts or bodily fluids can be obtained.

The strong correlation between PAM concentrations measured using the PAM-LIA assay and the amidating activity supports the assay's reliability as a proxy for PAM activity. The assay’s compatibility with various sample matrices, including EDTA plasma, serum, and Li-Heparin plasma, increases its versatility and utility in clinical testing. The broad operational temperature range (18–32 °C) of the PAM-LIA assay indicates its robustness and adaptability to varying laboratory conditions without significant impact on accuracy. The high ex vivo stability of the PAM analyte in plasma samples, with only a 4.3% reduction in concentration after six freeze–thaw cycles, suggests that the assay can be used with stored samples without substantial loss of accuracy.

Furthermore, the PAM-LIA assay demonstrated successful application in measuring PAM concentrations in plasma samples from rat, porcine and ape species, highlighting its potential for use in comparative and translational research involving different mammalian models. The detection of PAM in various tissue extracts, including porcine pituitary extract, soluble and solubilized membrane liver fraction from rat, suggests that the assay could detect different PAM isoforms expressed in various tissues.

Among the immunoassay-based methods for direct quantification of PAM, the PAM-LIA assay has shown several advantages when compared to previously reported methods. To our knowledge, Sturmer et al. was the first to report the direct quantification of PAM in an immunoassay-based setup, utilizing chicken antibodies to detect rat PAM^36^. However, it is important to note that although rat and human PAM share a high sequence similarity, the use of such an assay setup may not be the most suitable for detecting human PAM. Our results demonstrate a significant decline in antibody sensitivity when plasmatic rat PAM is used as an analyte.

Unlike PAM-LIA assay, the commercially available immunoassay-based kits for human PAM detection lack information regarding the correlation between PAM concentration and PAM activity (e. g. MyBioSource, Cat# MBS2024085), which raises questions about the validity of these assays for accurate PAM quantification. Additionally, the lack of information regarding the target of the solid- and liquid phase antibodies makes it challenging to distinguish whether the detected product is a single subunit or the full-length PAM. This ambiguity adds further uncertainty to the quantification process.

When comparing the analytical performance characteristics of different assay setups, it becomes evident that the PAM-LIA assay outperforms the other reported assay set-ups with respect to calibration range and range of operational temperature, demonstrates higher precision (intra- and inter-CV), reproducibility, linearity, spiking recovery and correlation with PAM α-amidating activity (Tab. S2). The wide calibration range of the PAM-LIA eliminates the need for sample dilution, which is required for previously reported assays. This advantage becomes especially significant when a large number of samples need to be analyzed. Another advantage of PAM-LIA assay is its one-step protocol, which enables the simultaneous incubation of the target analyte with detection and capture antibodies. In contrast, alternative immunoassays, such as the one described by Sturmer et al. or commercially available ELISA kits, involve more intricate time-consuming procedures that require preliminary antibody-antigen formation steps, sequential incubation of the analyte with capturing antibodies, followed by the washing steps, and subsequent incubation with detection antibodies.

The compatibility of the one-step PAM-LIA assay with high-throughput screening processes, such as 96-well format plates, makes it suitable for large-scale clinical automated testing and research. In a sub-cohort of 4850 individuals, we observed the correlation between PAM concentration and various clinical baseline parameters, including SBP and DBP, suggesting its potential for identifying subpopulations at risk for specific health conditions or monitoring intervention effectiveness.

Our study's findings support the potential use of PAM as a diagnostic and prognostic biomarker for various cardiovascular conditions, such as heart failure, atrial fibrillation, and major adverse cardiovascular events. These results align with prior studies indicating that patients with higher incidences of HF and AF tend to exhibit elevated PAM activity^28^. Our data supports the previously proposed hypothesis that the elevated PAM levels could have several origins. Firstly, the co-secretion of atrial natriuretic peptide (ANP) and brain natriuretic peptide (BNP), with PAM being involved in secretory vesicles formation in the heart atrium^29^. Secondly, the increased levels of bioactive adrenomedullin secretion, a peptide hormone that is known to be elevated in patients with HF, AF, and other cardiovascular pathologies, and which requires PAM for its activation. We have also observed significant correlation between PAM-LIA and bioactive adrenomedullin concentration in MPP sub cohort (Fig. S4).

Interestingly, with respect to diabetes our observations for PAM concentration correlated only partially with the data observed for PAM activity^28^. Unlike for PAM-AMA, there was no significant difference in PAM concentration measured in subjects with prevalent DM when compared with healthy individuals (Fig. S5A). This finding is rather surprising, as reduced PAM activity in diabetes has long been associated with several mutations in PAM^23–25,37^. However, our data suggests that in diabetes the concentration of PAM may be affected differently than its activity. In fact, we did not observe a significant negative correlation of PAM concentration with glucose levels (Fig. S5B), as was observed for PAM activity in subjects with prevalent diabetes. Furthermore, no correlation of PAM-LIA and glucose was found in subjects free of diabetes (Fig. S5C). The distinguish between PAM activity and concentration could shed more light on the role of PAM in diabetes development, but further investigation is needed to fully understand the underlying mechanisms.

In contrast to diabetes, PAM concentration was found to be reduced in patients with incident AD compared to healthy individuals, as measured in a sub-cohort of MPP (Fig. S6A). Furthermore, we observed that the decreased level of PAM served as prognostic indicator for the AD incidence, since reduction of PAM concentration was evident as early as 7 years prior to the AD diagnosis (Fig. S6B).

AD is a complex neurodegenerative process characterized by various features, including reduced cerebral blood flow and cognitive impairments. The most prominent hallmarks of AD are the extracellular accumulation of misfolded amyloid-ß and the intracellular deposition of hyperphosphorylated tau protein in the form of tangles. Several peptide hormones, such as adrenomedullin^38–40^, pituitary adenylate cyclase-activating peptide^41^, vasoactive intestinal peptide^41,42^, glucagon-like peptide 1^43^, neuropeptide Y^44^ etc., have been shown to effectively attenuate the symptoms associated with AD due to their neuroprotective properties and require α-amidation for their full biological activity. Considering this, the reduction in PAM concentration could be linked to the progression of clinical AD, as it may lead to a decrease in the production of active peptide hormones necessary for executing their neuroprotective function. This suggests that the impairment of PAM-mediated α-amidation could play a role in the pathogenesis of AD.

In conclusion, the PAM-LIA assay represents a robust, reliable, and versatile method for quantifying PAM concentrations in various sample matrices, with promising applications in clinical research and diagnostics. Future studies should focus on validating the assay in the context of specific disease conditions. The PAM-LIA assay provides a valuable tool for advancing the understanding of the physiological and pathological roles of PAM and its potential as a therapeutic target.

## Methods

### Design and heterologous expression of immunogen constructs

Soluble PHM and PAL subunits, comprising 31–377 residues (PHM_31–377_) and 494–817 residues (PAL_494–817_) of human PAM (UniProtKB: P19021-1), respectively, were synthesized and separately expressed in transiently transfected human kidney 293 (HEK293) cell. Both constructs were C-terminally truncated with decahistidine tag. The overexpressed recombinant constructs PHM_31–377_ and PAL_494–817_ were purified by nickel affinity chromatography with > 95% sample purity as measured by capillary gel electrophoresis, and used for immunization or screening of hybridoma cells.

### Antibody development

Monoclonal antibodies directed against soluble PAL and PHM subunit were generated by a standard fusion procedure between immunized Balb/c mice spleen cells with SP2/0 myeloma cells, describes elsewhere^45,46^. The resulting hybridoma cell lines were selected based on their ability to secret specific monoclonal antibodies against the respective immunogen. Finally, the antibodies were purified via protein A chromatography, yielding > 95% sample purity as measured by capillary gel electrophoresis.

### Chemiluminescence immunoassay for PAM quantification (PAM-LIA)

#### Solid- and liquid phase antibody

200 µL of 10 µg/mL α-PAL antibodies (2 µg/well) were dissolved in 50 mM Tris/HCl, 100 mM NaCl, pH 7.8 and applied to high-binding polystryrene microtiter plates per well and incubated for 18 h at 4–6 °C. Unbound α-PAL antibodies were discarded. After blocking with 6.5 mmol/L of KH_2_PO4, 3.5 mmol/L of K_2_HPO_4_, 3% Karion, 0.5% protease free BSA, pH 6.6–6.8, the coated MTP were air dried for 20 h and stored at 4–6 °C.α-PHM antibodies were incubated with a 1:3 molar ratio of methylacridinium *N*-hydroxysuccinimide ester (MACN) (1 g/L; Invent diagnostic GmbH) for 30 min at 20 °C in the dark. After quenching the reaction with 5% 1M Tris/HCl, pH 7.0, the MACN-labeled α-PHM antibodies were separated from the unbound MACN by gel filtration via CentriPure P10 column (emp BIOTECH GmbH) and further purified using HPLC column (Knauer, 0.5 mL/min flow rate). After supplementation with 5% BSA, the labeled antibodies were stored at − 20 °C.

#### Calibration

The assay was calibrated with defined concentrations of commercially obtained recombinant PAM (SinoBiological; cat-no.: 13624-H08H) in a range of 0–723.4 ng/mL, dissolved in 100 mM Tris/HCl, pH 8.0, 3% protease free BSA.

#### Standard assay protocol

20 µL of EDTA plasma sample/calibrator in duplicates and 200 µL assay buffer (115 mM KH_2_PO_4_, 185 mM K_2_HPO_4_, 100 mM NaCl, 10 mM EDTA, 0.1% w/v unspecified bovine IgG, 0.02% w/v unspecified murine IgG, 50 mM amastatin HCl, 100 µM leupeptin hemisulfate, 0,5% protease free BSA, pH 7,0), containing 50 ng/mL liquid phase antibodies (10 ng/well), were applied to a microtiter 96-well plate (Greiner Bio-One International AG), coated with solid phase antibodies. The incubation for 3 h at 22 °C under agitation of 600 rpm followed (Titramax 101, Heidolph Instruments GmbH & CO. KG). Unbound labeled antibodies were removed by washing 5 times with 350 µL standard washing buffer (sphingotec GmbH). Chemiluminescence emission was detected for 1 s with Centro LB 960 microtiter plate luminescence reader (Berthold Technologies).

#### Assay performance study

The inter-assay precision was determined from twelve EDTA plasma samples, comprising seven samples with different concentration spectrum of endogenous PAM (range 0.65–15.6 ng/mL) and five samples spiked with recombinant PAM (range 51.1–305.9 ng/mL). Prior the measurements samples were subdivided into aliquots and stored at − 80 °C. The measurements were performed over the period of 5 days with three runs per day in a total of 15 independent assays, conducted by three operators. For one run one microtiter plate was used for calibrators and samples, each in duplicates. The limit of quantification (LoQ), defined as the concentration of PAM that can be reliably measured with a coefficient of variation (CV) of 20%, was determined using GraphPad Prism 8.4.3. To this end, twelve EDTA plasma samples were selected, covering a concentration range of 75.3 pg/mL to 305.9 ng/mL. Each sample was measured in duplicates by three different operators in 15 independent assays. The CV (%) was calculated for each measurement and plotted against the corresponding PAM concentration. The LoQ value was then determined by interpolating from dose–response curve and represented the PAM concentration at which the CV reached 20%. The limit of detection (LoD) was determined by Analyse-it software, utilizing 55 independent measurements of plasma samples depleted from PAM (limit of blank, LoB), and 32 samples with a low analyte concentration ranging between 114 and 308 pg/mL. Nonparametric estimation of normal quantile with an error of 5% was used (Fig. S7).

The intra-assay precision was determined by concentration measurement of three EDTA samples with PAM concentration of 1.4 ng/mL, 66 ng/mL and 117 ng/mL. PAM concentration in each sample was determined as 16-times iteration within of a single assay.

Spiking recovery (accuracy) was assessed by adding known concentrations (50 ng/mL, 100 ng/mL, 250 ng/mL, and 500 ng/mL) of synthetic full-length PAM (SinoBiological) to the analyte-depleted EDTA plasma (zero-matrix) and comparing the detected PAM level to expected concentrations. Each concentration was measured in quadruplicate. To generate zero-matrix, EDTA plasma was incubated with α-PAL and α-PHM antibodies, coupled to a ultralink hydrazine resin (ThermoFischer Scientific), for 4 h at 4–6 °C. The remaining PAM concertation in the analyte free matrix was < 250 pg/mL, when measured in PAM-LIA assay under standard protocol conditions.

For dilution recovery (linearity), three pools of EDTA plasma were 10% stepwise diluted with analyte-low matrix. Plasma pool concentrations were 674.7 ng/mL and 323.5 ng/mL, when spiked with recombinant PAM, and 91.2 ng/mL, containing the endogenous PAM of several self-reported healthy individuals. The zero-matrix was generated as described in spiking recovery experiments.

For mixing recovery, eight EDTA plasma samples with predetermined PAM concentration in a range between 1.7 and 481.1 ng/mL were mixed in a 1 to 1 ratio in random manner, resulting in total of 19 combined pools. The deviation between the expected and measured PAM concentration was determined.

To access the substrate interference, two EDTA plasma pools (low EDTA plasma pool with 54.7 ng/mL endogenous PAM; high EDTA plasma pool spiked with 365 ng/mL recombinant PAM) were mixed with 32 substances in a significant molar excess in respect to analyte (Tab. S1), incubated for 1 h at RT and measured according to a standard assay protocol. The samples were measured in quadruplicate. Recovery calculations were corrected for the effect of volume increase after supplementation and deviations to the not supplemented samples were calculated.

To determine the freeze–thaw stability under ex vivo conditions, the six EDTA-plasma samples from self-reported healthy individuals underwent up to 6 freeze–thaw cycles. The samples were stored at − 80 °C for at least 24 h prior the following freeze–thaw cycle to occur.

To access the operative temperature range, eight EDTA plasma samples in a range between 1,8 ng/mL to 466.4 ng/mL were measured in duplicate according to a standard assay protocol at 18 °C, 20 °C, 22 °C, 25 °C, 28 °C, 32 °C. The PAM concentration determined at 22 °C was set as a reference point.

The acceptance criterion for all technical assay parameters was ± 20% CV from the original or expected concentration.

#### Correlation studies

To determine the matrix effect on PAM concentration, PAM-LIA was measured in matched EDTA plasma and serum samples, as well as in matched EDTA plasma and Li-Heparin samples from 31 and 70 healthy self-reported individuals, respectively. Additionally, PAM amidating activity were measured in Li-Heparin samples, in order to investigate the correlation between the soluble PAM activity and PAM concertation. PAM activity was measured in house as described in Kaufmann et al.^28^. Correlations were calculated as Spearman rank correlation. *p* values < 0.05 were considered significant.

#### MPP study population

For normal distribution and other baseline characteristics of PAM concentration in healthy population, the sub cohort of randomly selected 4850 individuals from the Swedish single-center prospective population based study (Malmö Preventive Project, MPP^47^) were measured in serum. Previously, detailed descriptions were provided for the methods used to assess baseline cardiovascular risk factors and for linking records with the Swedish national inpatient and cause of death registries to retrieve endpoints related to the incidence of major adverse cardiovascular events (MACE), atrial fibrillation (AF) and congestive heart failure (HF)^48–50^. Written informed consent was obtained from all participants and/or their legal guardians before entering MPP-RES. The study was approved by The Regional Ethical Review board at Lund University, Sweden (LU 244-02) and complied with the Helsinki Declaration (IRB = 2009/633).

The baseline levels of PAM-LIA in the MPP were expressed in quartiles, with quartile 1, 2, 3 and 4 comprising the PAM-LIA values of 9.5–67.1 ng/mL, 67.2–79.5 ng/mL, 79.6–93.0 ng7mL, 93.1–242.2 ng/mL, respectively. Multiple comparisons of baseline clinical characteristics (e.g. age, gender, LDL, HDL, cholesterol, glucose etc.) according to PAM quartiles were performed using one-way ANOVA. In order to assess the incidence of HF, AF, and MACE during the 16-year follow-up period with respect to PAM concentration, the Cox Proportional Hazards Model was used. The individuals with prevalent coronary event and stroke were excluded. The model was adjusted for baseline risk factors including age, gender, BMI, systolic blood pressure, diastolic blood pressure, glucose, diabetes, LDL, HDL, total cholesterol, and triglycerides. The incidence of Alzheimer´s disease was accessed during the 7-year follow-up period in MPP sub-cohort, comprising n = 3886 individuals (Cox Proportional Hazards Model). The individuals with prevalent AD were excluded. The cut-off value of 88.2 ng/mL were used. The model was adjusted for baseline risk factors as described above.

### Statistical analysis

All statistical analysis were carried out with GraphPrism 8.4.3 and Analyse-it software. Graphics were prepared using Chimera^51^. Distribution was tested with the D'Agostino-Pearson omnibus test.

## Supplementary Information

Supplementary Information.

## Data Availability

The datasets used and/or analysed during the current study available from the corresponding author on reasonable request.

## References

[1] Eipper BA, Stoffers DA, Mains RE (1992). The biosynthesis of neuropeptides: Peptide alpha-amidation. Annu. Rev. Neurosci..

[2] Eipper, B. A., Mains, R. E., & Street, N. W. “PEPTIDE a-AMIDATION,” 1988.

[3] Kumar D, Mains RE, Eipper BA (2016). From POMC and α-MSH to PAM, molecular oxygen, copper, and vitamin C. J. Mol. Endocrinol..

[4] Merkler DJ, Hawley AJ, Eipper BA, Mains RE (2022). Peptidylglycine α-amidating monooxygenase as a therapeutic target or biomarker for human diseases. Br. J. Pharmacol..

[5] Owen TC, Merkler DJ (2004). A new proposal for the mechanism of glycine hydroxylation as catalyzed by peptidylglycine α-hydroxylating monooxygenase (PHM). Med. Hypotheses.

[6] Prigge ST, Eipper BA, Mains RE, Amzel LM (2004). Dioxygen binds end-on to mononuclear copper in a precatalytic enzyme complex. Science (80-).

[7] Chufán EE, De M, Eipper BA, Mains RE, Amzel LM (2009). Amidation of bioactive peptides: The structure of the lyase domain of the amidating enzyme. Structure.

[8] Gaier ED (2014). Genetic determinants of amidating enzyme activity and its relationship with metal cofactors in human serum. BMC Endocr. Disord..

[9] Perkins SN, Husten EJ, Eipper BA (1990). The 108-kDa peptidylglycine α-amidating monooxygenase precursor contains two separable enzymatic activities involved in peptide amidation. Biochem. Biophys. Res. Commun..

[10] Murthy ASN, Mains RE, Eipper BA (1986). Purification and characterization of peptidylglycine α-amidating monooxygenase from bovine neurointermediate pituitary. J. Biol. Chem..

[11] Mehta NM, Gilligan JP, Jones BN, Bertelsen AH, Roos BA, Birnbaum RS (1988). Purification of a peptidylglycine α-amidating enzyme from transplantable rat medullary thyroid carcinomas. Arch. Biochem. Biophys..

[12] Gilligan JP, Lovato SJ, Mehta NM, Bertelsen AH, Jeng AY, Tamburini PP (1989). Multiple forms of peptidyl α-amidating enzyme: Purification from rat medullary thyroid carcinoma CA-77 cell-conditioned medium. Endocrinology.

[13] May V, Cullen EI, Braas KM, Eipper BA (1988). Membrane-associated forms of peptidylglycine alpha-amidating monooxygenase activity in rat pituitary. Tissue specificity. J. Biol. Chem..

[14] Czyzyk TA (2005). Deletion of peptide amidation enzymatic activity leads to edema and embryonic lethality in the mouse. Dev. Biol..

[15] Kolhekar AS (1997). Neuropeptide amidation in drosophila: Separate genes encode the two enzymes catalyzing amidation. J. Neurosci..

[16] Iliadi KG (2008). nemy encodes a cytochrome b561 that is required for Drosophila learning and memory. Proc. Natl. Acad. Sci. U. S. A..

[17] Schafer MKH, Stoffers DA, Eipper BA, Watson SJ (1992). Expression of peptidylglycine α-amidating monooxygenase (EC 1.14.17.3) in the rat central nervous system. J. Neurosci..

[18] Bolkenius FN, Ganzhorn AJ (1998). Peptidylglycine α-amidating mono-oxygenase: Neuropeptide amidation as a target for drug design. Gen. Pharmacol..

[19] Gether U, Aakerlund L, Schwartz TW (1991). Comparison of peptidyl-glycine α-amidation activity in medullary thyroid carcinoma cells, pheochromocytomas, and serum. Mol. Cell. Endocrinol..

[20] Wand GS, Ney RL, Stephen B, Eipper BA, Mains RE (1985). Characterization of peptide alpha-amidation activity in human plasma and tissues. Metabolism.

[21] Tsukamoto T, Kayama H, Watanabe T, Yamamoto T, Noguchi M, Asoh T (1995). Increased peptidylglycine α-amidating monooxygenase activity in cerebrospinal fluid of patients with multiple sclerosis. Intern. Med..

[22] Gonzalez H (2009). Identification of novel candidate protein biomarkers for the post-polio syndrome: Implications for diagnosis, neurodegeneration and neuroinflammation. J. Proteom..

[23] Thomsen SK (2018). Type 2 diabetes risk alleles in PAM impact insulin release from human pancreatic β-cells. Nat. Genet..

[24] Chen YC (2020). PAM haploinsufficiency does not accelerate the development of diet- and human IAPP-induced diabetes in mice. Diabetologia.

[25] Sheng B, Wei H, Li Z, Wei H, Zhao Q (2022). PAM variants were associated with type 2 diabetes mellitus risk in the Chinese population. Funct. Integr. Genomics.

[26] Wand GS, May C, May V, Whitehouse PJ, Rapoport SI, Eipper BA (1987). Alzheimer’s disease: Low levels of peptide alpha-amidation activity in brain and CSF. Neurology.

[27] Kaufmann, P. *et al.*, Role of adrenomedullin processing in development of Alzheimer’s Disease. Poster presented at the LANCET Summit, 2021, Presymptomatic Prevention and Treatment of Neurodegenerative Diseases.

[28] Kaufmann P, Bergmann A, Melander O (2021). Novel insights into peptide amidation and amidating activity in the human circulation. Sci. Rep..

[29] Bäck N, Luxmi R, Powers KG, Mains RE, Eipper BA (2020). Peptidylglycine α-amidating monooxygenase is required for atrial secretory granule formation. Proc. Natl. Acad. Sci. U. S. A..

[30] Miyazaki N, Uemura T (1991). Determination of peptidylglycine α-amidating monooxygenase activity in human serum by thin-layer chromatography. Anal. Biochem..

[31] Rocchi P (2001). Expression of adrenomedullin and peptide amidation activity in human prostate cancer and in human prostate cancer cell lines. Cancer Res..

[32] Treston AM (1993). Biochemical characterization of peptide alpha-amidation enzyme activities of human neuroendocrine lung cancer cell lines. Cell Growth Differ..

[33] Noguchi M, Takahashi K, Okamoto H (1988). Characterization of peptidylglycine alpha-amidating activities in rat pituitary, brain and small intestine using glycine-extended C-terminal analogues of vasoactive intestinal polypeptide as substrate. Tohoku J. Exp Med..

[34] Tajima M (1990). The reaction product of peptidylglycine alpha-amidating enzyme is a hydroxyl derivative at alpha-carbon of the carboxylterminal glycine. J. Biol. Chem..

[35] Vaeruy H, Nyberg F, Franzen H, Terenius L (1987). Characterization of a substance P-Gly amidating enzyme in human cerebrospinal fluid. Biochem. Biophys. Res. Commun..

[36] Sturmer AM, Driscoll DP, Jackson-Matthews DE (1992). A quantitative immunoassay using chicken antibodies for detection of native and recombinant α-amidating enzyme. J. Immunol. Methods.

[37] Umapathysivam, M. M., Araldi, E., Hastoy, B., & Dawed, A. Y. Type 2 diabetes risk alleles in peptidyl-glycine alpha-amidating monooxygenase influence GLP-1 levels and response to GLP-1 receptor agonists Corresponding Author : T2D-risk,” (2023). 10.1186/s13073-026-01630-0 PMC13072570 41906117

[38] Honda M (2006). Adrenomedullin improves the blood-brain barrier function through the expression of claudin-5. Cell. Mol. Neurobiol..

[39] Hippenstiel S (2002). Adrenomedullin reduces endothelial hyperpermeability. Circ. Res..

[40] Larrayoz IM, Ferrero H, Martisova E, Gil-Bea FJ, Ramírez MJ, Martínez A (2017). Adrenomedullin contributes to age-related memory loss in mice and is elevated in aging human brains. Front. Mol. Neurosci..

[41] Solés-Tarrés I, Cabezas-Llobet N, Vaudry D, Xifró X (2020). Protective effects of pituitary adenylate cyclase-activating polypeptide and vasoactive intestinal peptide against cognitive decline in neurodegenerative diseases. Front. Cell. Neurosci..

[42] Korkmaz OT, Ay H, Aytan N, Carreras I, Kowall NW (2018). Vasoactive intestinal peptide decreases β-amyloid accumulation and prevents brain Atrophy in the 5xFAD mouse model of Alzheimer ’ s disease. J. Mol. Neurosci..

[43] Reich N, Hölscher C (2022). The neuroprotective effects of glucagon-like peptide 1 in Alzheimer’s and Parkinson’s disease: An in-depth review. Front. Neurosci..

[44] Chen X, Du Y, Chen L (2019). Neuropeptides exert neuroprotective effects in Alzheimer’s disease. Front. Mol. Neurosci..

[45] Lane RD (1985). A short-duration polyethylene glycol fusion technique for increasing production of monoclonal antibody-secreting hybridomas. J. Immunol. Methods.

[46] Hikawa N, Takenaka T (1997). Method for production of neuronal hybridoma using emetine and actinomycin D. Brain Res. Protoc..

[47] Berglund G (2000). Long-term outcome of the malmo preventive project: Mortality and cardiovascular morbidity. J. Intern. Med..

[48] Smith JG (2010). Assessment of conventional cardiovascular risk factors and multiple biomarkers for the prediction of incident heart failure and atrial fibrillation. J. Am. Coll. Cardiol..

[49] Smith JG, Platonov PG, Hedblad B, Engström G, Melander O (2010). Atrial fibrillation in the Malmö diet and cancer study: A study of occurrence, risk factors and diagnostic validity. Eur. J. Epidemiol..

[50] Tasevska I, Enhörning S, Persson M, Nilsson PM, Melander O (2016). Copeptin predicts coronary artery disease cardiovascular and total mortality. Heart.

[51] Pettersen EF (2004). UCSF Chimera: A visualization system for exploratory research and analysis. J. Comput. Chem..

