# Supplementary material for: Immunoassay-based quantification of full-length peptidylglycine alpha-amidating monooxygenase in human plasma
Source: Sci Rep. 2023 Jul 4;13:10827. doi: 10.1038/s41598-023-37976-3 (PMC10319883; doi:10.1038/s41598-023-37976-3)
Supplement: Supplementary file 1 — Supplementary Information. [file 41598_2023_37976_MOESM1_ESM.docx]

Supplementary Information for the Scientific Reports Manuscript:

**Immunoassay-based quantification of full-length peptidylglycine alpha-amidating monooxygenase in human plasma**

**
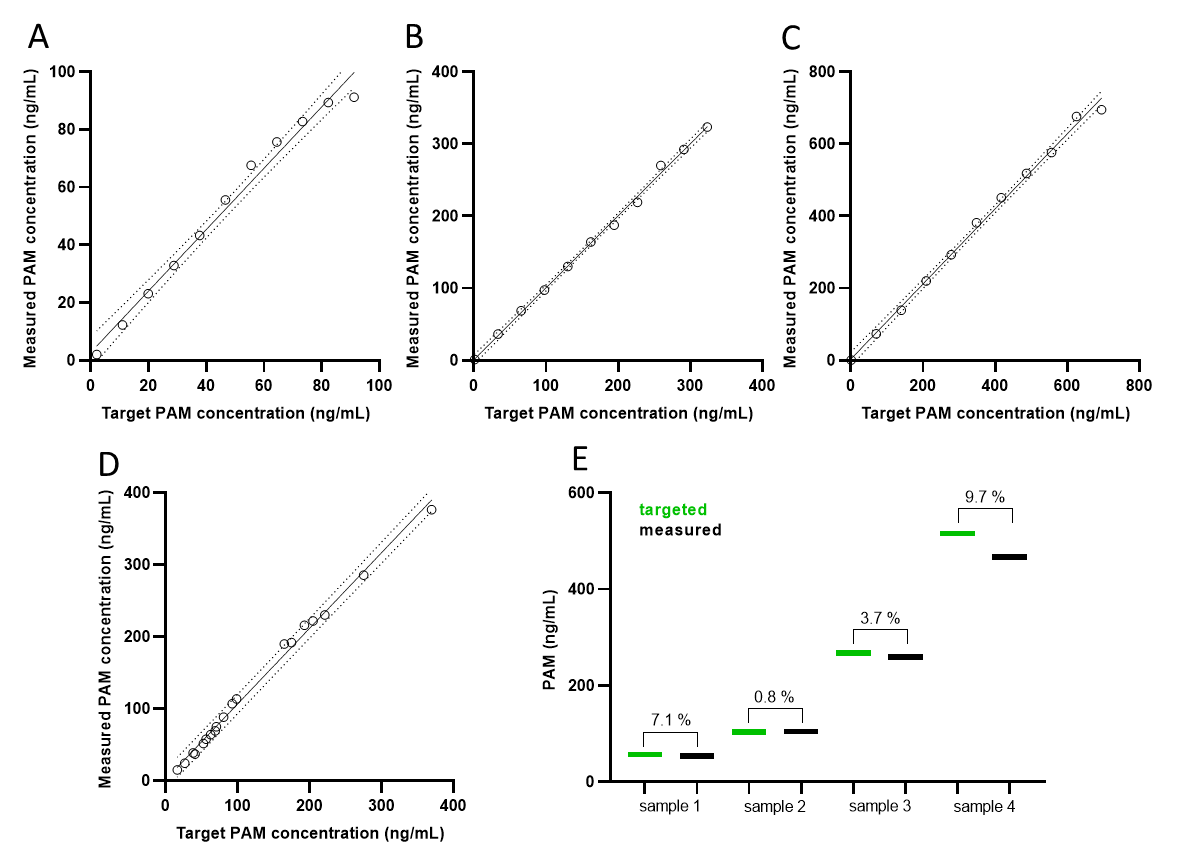
**

**Supplemental Figure S1**: (A-C) Assay linearity accessed by dilutional recovery with starting PAM concentration of 91.2 ng/mL (A), 323.5 ng/mL (B) and 674.7 ng/mL (C); (D) Mixing recovery of n=19 total sample pools with PAM concentrations ranging from 1.7 ng/mL to 481.1 ng/mL. The 95% confidence interval is shown as dotted lines; (E) The accuracy of PAM-LIA (spiking recovery), measured in four analyte-depleted samples, spiked with 50 ng/mL (sample 1), 100 ng/mL (sample 2), 250 ng/mL (sample 3), and 500 ng/mL (sample 4). The deviation between the targeted and measured PAM concentration are shown in % (CV).


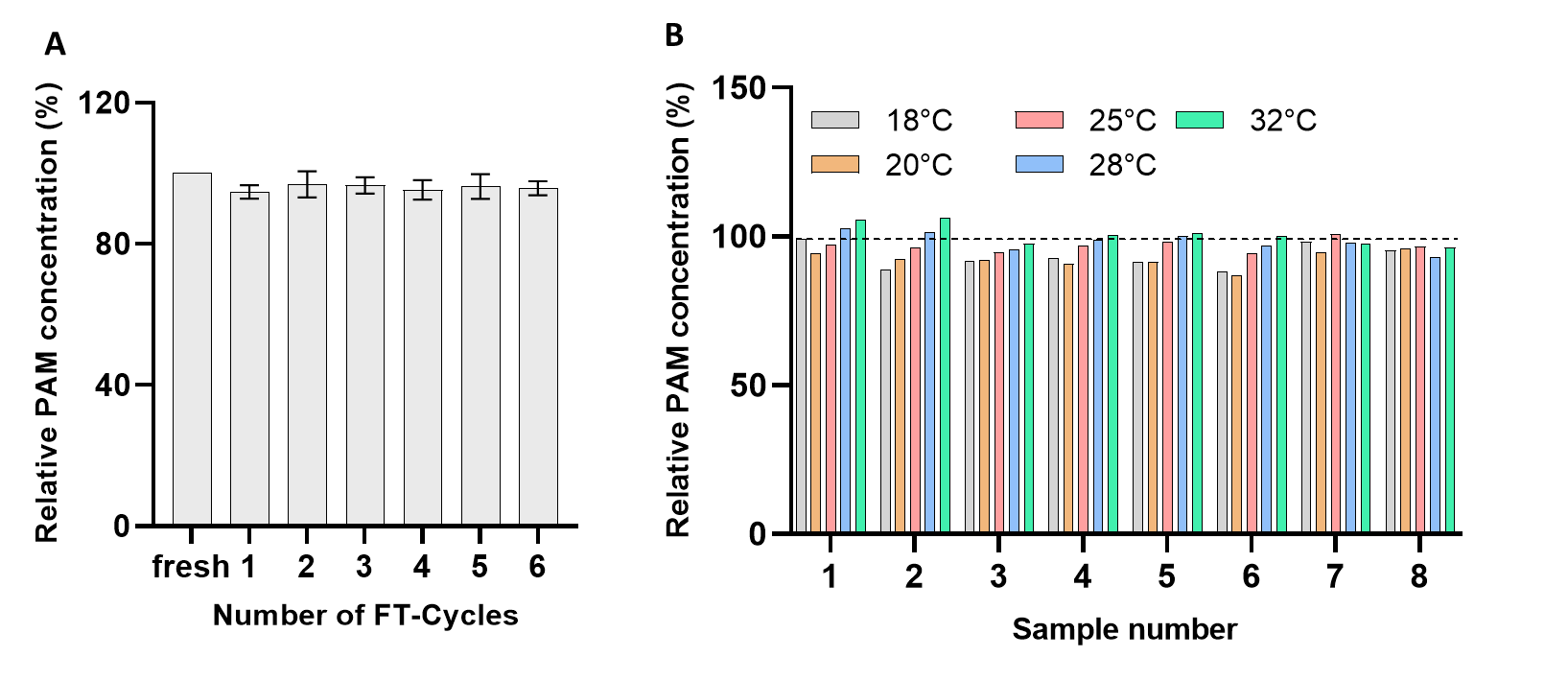


**Supplemental Figure S2**: (A) Freeze-thaw stability of the native PAM. Each bar represents the relative concentration of PAM, averaged over 6 measurements. The concentration of the unfrozen sample was set as 100%. (B) Operative temperature range of PAM-LIA assay. Each bar represents a relative PAM concentration determined in duplicate in eight EDTA plasma samples with increasing PAM concentration, ranging between 1.8 ng/mL to 466.4 ng/mL. The PAM concentration determined at 22°C was set as 100%.


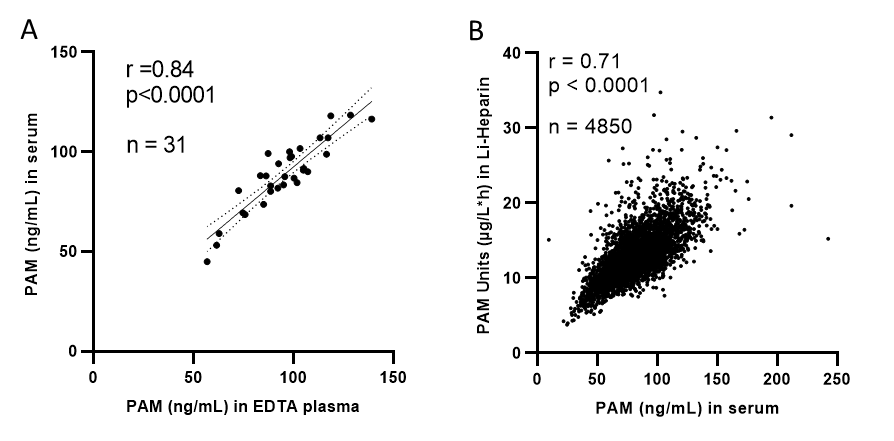


**Supplemental Figure S3**: Non-parametric Spearman correlation of PAM concentration, measured between matched EDTA plasma and serum samples in PAM-LIA assay (A). The correlation was also determined between PAM concentration (in ng/mL) and PAM amidating activity (in µg/L*h) in large population-based cohort, measured as described in Kaufmann et al. 2021. PAM concentration and activity was determined in matched serum and Li-Heparin samples, respectively. n being the number of matched pairs, r representing the correlation coefficient and p-value for statistical significance. The 95% confidence interval is shown as dotted lines.

**Supplemental Figure S4**: Spearman rank correlations between PAM-LIA and bioactive adrenomedullin levels, measured in MPP subcohort of 4530 individuals, with r being the correlation coefficients and p indicating the statistical significance.


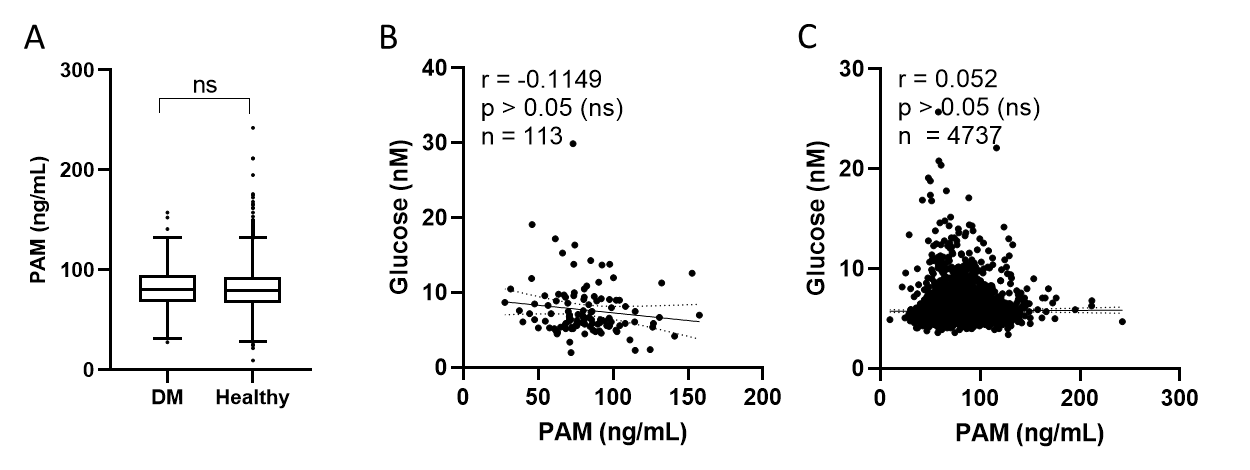


**Supplemental Figure S5**: PAM concentration and correlation with glucose levels in subjects with prevalent diabetes (DM) and healthy individuals. (A) A box plot of PAM concentration (PAM-LIA) in the study cohort, with 113 subjects with prevalent diabetes and 4737 healthy individuals. The non-parametric Mann-Whitney test was used to test for significance, with n being the number of pairs. Spearman rank correlations between PAM-LIA and glucose levels in subjects with prevalent diabetes (B) and healthy individuals (C), respectively, with r being the correlation coefficients and p indicating the statistical significance.


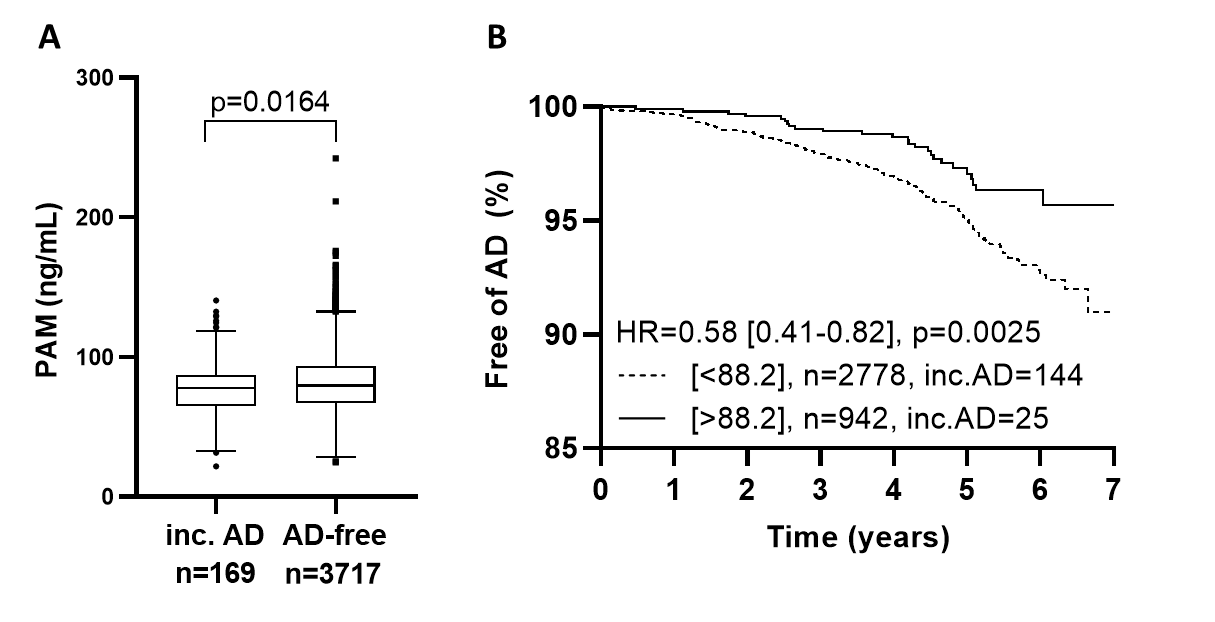


**Supplemental Figure S6**: PAM concentration in subjects with incident AD (inc. AD) and healthy individuals (AD-free). (A) Median of PAM concentration (in ng/mL), compared between AD-free and incident AD group. (B) Kaplan-Meier survival analysis over the follow-up time of 7 years, measured in MPP subcohort. For PAM concentration first and second group represented the cutoff values lower or higher than 88.2 ng/mL, respectively. HR for hazard ratio and n for number of individuals.

**Supplemental Figure S7**: Limit of detection of PAM-LIA assay. LoB comprised 55 independent measurements of PAM-depleted plasma samples and 32 samples with low PAM concentration in a range between 114 pg/mL and 308 pg/mL.

**Supplemental Table S1**: Substance interference study

|  | Final concentration | Low pool | | High pool | |
| --- | --- | --- | --- | --- | --- |
|  |  | PAM (ng/mL) | Recovery (%) | PAM (ng/mL) | Recovery (%) |
| no supplemented |  | 54.7 |  | 364.6 |  |
| H_2_O |  | 51.6 | 100.0 | 347.5 | 100 |
| Epinephrine | 12 µg/mL | 51.0 | 98.7 | 340.2 | 97.9 |
| Trisodium Citrate | 165 mg/mL | 51.1 | 99.0 | 340.0 | 97.8 |
| Phenylephrine | 6 µg/mL | 50.3 | 97.5 | 339.5 | 97.7 |
| L-Ascorbic Acid | 1.05 mg/mL | 50.7 | 98.3 | 325.5 | 93.7 |
| Thiotropium | 0.96 pg/mL | 50.8 | 98.4 | 334.0 | 96.1 |
| Capropril | 58 µg/mL | 49.7 | 96.2 | 331.2 | 95.3 |
| Gentamicin Sulfate | 0.6 mg/mL | 49.4 | 95.6 | 335.4 | 96.5 |
| Hemoglobin | 200 mg/mL | 52.0 | 100.7 | 311.5 | 89.6 |
| Fentanyl | 6 µg/mL | 49.4 | 95.8 | 327.5 | 94.2 |
| Furosimide | 0.32 mg/mL | 48.7 | 94.4 | 326.9 | 94.1 |
| Salicylic acid | 572 µg/mL | 46.1 | 89.3 | 315.4 | 90.8 |
| Acetyl salicylic acid | 0.6 mg/mL | 46.8 | 90.6 | 319.7 | 92.0 |
| Acetamiophen | 3.12 mg/mL | 46.0 | 89.1 | 318.6 | 91.7 |
| Ibuprofen | 4.38 mg/mL | 46.1 | 89.4 | 323.1 | 93.0 |
| Dexamethasone | 0.24 mg/mL | 46.4 | 89.9 | 317.4 | 91.3 |
| Dextrometrophan | 3.12 µg/mL | 49.2 | 95.3 | 338.7 | 97.5 |
| Dobutamine | 24.2 µg/mL | 45.7 | 88.5 | 338.2 | 97.3 |
| Loratadine | 1.74 µg/mL | 46.4 | 89.9 | 337.0 | 97.0 |
| Nicardipine | 0.93 µg/mL | 46.7 | 90.4 | 337.0 | 97.0 |
| Valsatran | 0.234 mg/mL | 47.5 | 92.1 | 337.7 | 97.2 |
| Wasfarin | 1.5 mg/mL | 45.5 | 88.2 | 337.1 | 97.0 |
| Ionexol | 20 mg/mL | 48.9 | 94.7 | 346.9 | 99.8 |
| Sacubitril | 0.183 mg/mL | 49.2 | 95.4 | 343.6 | 98.9 |
| Sacubitrilat | 0.183 mg/mL | 49.3 | 95.6 | 344.4 | 99.1 |
| Vancomycin | 2 mg/mL | 48.2 | 93.4 | 332.1 | 95.6 |
| D-Glucose | 200 mg/mL | 49.3 | 95.5 | 344.5 | 99.1 |
| Bilirubin conjugated | 8 mg/mL | 47.9 | 92.7 | 326.1 | 93.8 |
| Bilirubin | 8 mg/mL | 47.6 | 92.2 | 331.6 | 95.4 |
| MeOH | 4.5 % (v/v) | 48.2 | 93.4 | 339.9 | 97.8 |
| DMSO | 4.5 % (v/v) | 47.7 | 92.5 | 338.3 | 97.4 |
| EtOH | 4.5 % (v/v) | 50.6 | 98.0 | 341.6 | 98.3 |

**Supplemental Table S2**: Technical characteristics comparison: previously reported and commercially available immunoassay-based methods for PAM quantification vs. PAM-LIA. n.d. – not determined

|  | **PAM-LIA assay** | **Quantitative immunoassay using chicken ab, Sturmer et al. 1992** | **ELISA Kit (Human PAM)** |
| --- | --- | --- | --- |
| Calibration range | 0.25 - 723.4 ng/mL | 0.78 - 12.5 ng/mL | 0.031 - 2 ng/mL |
| Sample volume (µL) | 20 (non-diluted) | 100 (diluted) | 10 (diluted) |
| Total Incubation time | 3h: single-step protocol | 3h 20 min: multi-step protocol | 3h: multi-step step protocol |
| Incubation temperature | 18 °C - 32 °C | 37 °C | 37 °C |
| Detection type | Chemiluminescence | Colorimetric | Colorimentic |
| Assay sensitivity | 189 pg/mL | 200 pg/mL | 13.9 pg/mL |
| Intra-assay CV | 2.2 % [1.3 % - 3.8 %] | 6.7 % [6.0 % - 7.6 %] | < 10 % |
| Inter-assay CV | 6.7 % [2.8 % - 12.3 %] | 8.7 % [7.0 % - 10.8 %] | < 12 % |
| Linearity in EDTA plasma | 96.5 % [86.6 % - 109.5 %] | n.d. | 88 % [78 % - 97 %] |
| Spiking recovery | 94.7% [90.3% - 99.2%] | n.d. | 93 % [85 % - 99 %] |
| Correlation with amidating activity | Spearman r = 0.98, p<0.0001, (n= 70) | Spearman r* = 0.89, p= 0.033, (n= 6) | n.d. |
| Specificity | Human, ape, porcine and rat | Rat | Human |
| Described in | present study | 10.1016/0022-1759(92)90053-V | commercially available; Cat: MBS2024085 |

* The nonparametric two-tailed Spearman correlation (GraphPad Prism) was calculated by correlating the concentration of enzyme determined by the immunoassay to the concentration of enzyme determined by the dansyl-activity assay, using the values from Fig. 3 of Sturmer et al., 1992.
